# Pathways for the progression of neurodegeneration in the dementia with Lewy bodies continuum

**DOI:** 10.1002/alz70856_102830

**Published:** 2025-12-24

**Authors:** Annegret Habich, Bastiaan G J van Tol, Milan Nemy, Michael J Firbank, John‐Paul Taylor, Eric Westman, Daniel Ferreira

**Affiliations:** ^1^ Division of Clinical Geriatrics, Center for Alzheimer Research, Department of Neurobiology, Care Sciences and Society, Karolinska Institutet, Stockholm, Sweden; ^2^ Division of Clinical Geriatrics, Center for Alzheimer Research, Department of Neurobiology, Care Sciences and Society, Karolinska Institute, Stockholm, Stockholms län, Sweden; ^3^ Division of Clinical Geriatrics, Center for Alzheimer Research, Department of Neurobiology, Care Sciences and Society, Karolinska Institutet, Stockholm, Stockholms län, Sweden; ^4^ Czech Technical University in Prague, Prague, Czech Republic; ^5^ Newcastle University, Newcastle upon Tyne, Newcastle, United Kingdom; ^6^ Translational and Clinical Research Institute, Newcastle University, Newcastle upon Tyne, United Kingdom; ^7^ Division of Clinical Geriatrics, Centre for Alzheimer Research, Karolinska Institutet, Stockholm, Sweden

## Abstract

**Background:**

While patients along the dementia with Lew bodies (DLB) continuum show widespread neurodegeneration, the determinants of this regional neurodegeneration remain largely unexplored. The underlying alpha‐synuclein pathology is hypothesized to spread along structural connections with affected brain regions undergoing neurodegeneration. We aimed to determine whether brain regions that are more closely connected to amygdala and brainstem, i.e. two suggested origins of the alpha‐synuclein pathology, exhibit greater volume reductions compared to more distantly connected regions. Additionally, we investigated whether regions that are more integrated in the brain network overall exhibit greater volume reductions compared to less integrated regio**ns**.

**Method:**

We included 80 patients along the DLB continuum and 65 cognitively healthy controls (HCs) from the Newcastle cohort of the European DLB consortium. Volumetric differences between DLB patients and HCs were quantified in 122 brain regions from T1‐weighted MRI, using averaged w‐scores. Tractography of white matter pathways between brain regions was performed on diffusion‐weighted images from DLB patients to build an average structural network. The distance of brain regions to amygdala and brain stem, and the regions’ integration in the network were calculated using graph theoretical network measures. Lastly, we correlated network measures with regional volumetric w‐scores.

**Result:**

Most brain regions showed lower volumes in patients along the DLB continuum compared with HCs, with neurodegeneration being most pronounced in temporal and parietal lobes. Regions more closely connected to the amygdala showed more volume reductions. In contrast, distance to the brainstem did not significantly correlate with regional w‐scores. Likewise, the integration of brain regions within the structural network did not significantly correlate with regional w‐scores.

**Conclusion:**

Our results emphasize that structural connections with the amygdala predict regional neurodegeneration, suggesting that the amygdala is an important relay region for the spread of the alpha‐synuclein pathology. Moreover, our findings support the use of imaging biomarkers to track disease progression in the absence of a topographic biomarker for the alpha‐synuclein pathology.

